# A meta-analysis of gene expression data highlights synaptic dysfunction in the hippocampus of brains with Alzheimer’s disease

**DOI:** 10.1038/s41598-020-64452-z

**Published:** 2020-05-20

**Authors:** Saeedeh Hosseinian, Ehsan Arefian, Hassan Rakhsh-Khorshid, Mehdi Eivani, Ameneh Rezayof, Hamid Pezeshk, Sayed-Amir Marashi

**Affiliations:** 1 Department of Biotechnology, College of Science, University of Tehran, Tehran, Iran; 2 Department of Microbiology, School of Biology, College of Science, University of Tehran, Tehran, Iran; 3 Pediatric Cell Therapy Research Center, Tehran University of Medical Sciences, Tehran, Iran; 4 Department of Biochemistry, Faculty of Biological Sciences, Tarbiat Modares University, Tehran, Iran; 5 Neuroscience Lab, Department of Animal Biology, School of Biology, College of Science, University of Tehran, Tehran, Iran; 6 Neuroscience Lab, Department of Animal Biology, School of Biology, College of Science, University of Tehran, Tehran, Iran; 7 School of Mathematics, Statistics and Computer Science, College of Science, University of Tehran, Tehran, Iran; 8 School of Biological Sciences, Institute for Research in Fundamental Sciences (IPM), Tehran, Iran

**Keywords:** Animal disease models, Microarray analysis, Alzheimer's disease, Hippocampus, Synaptic plasticity

## Abstract

Since the world population is ageing, dementia is going to be a growing concern. Alzheimer’s disease is the most common form of dementia. The pathogenesis of Alzheimer’s disease is extensively studied, yet unknown remains. Therefore, we aimed to extract new knowledge from existing data. We analysed about 2700 upregulated genes and 2200 downregulated genes from three studies on the CA1 of the hippocampus of brains with Alzheimer’s disease. We found that only the calcium signalling pathway enriched by 48 downregulated genes was consistent between all three studies. We predicted miR-129 to target nine out of 48 genes. Then, we validated miR-129 to regulate six out of nine genes in HEK cells. We noticed that four out of six genes play a role in synaptic plasticity. Finally, we confirmed the upregulation of miR-129 in the hippocampus of brains of rats with scopolamine-induced amnesia as a model of Alzheimer’s disease. We suggest that future research should investigate the possible role of miR-129 in synaptic plasticity and Alzheimer’s disease. This paper presents a novel framework to gain insight into potential biomarkers and targets for diagnosis and treatment of diseases.

## Introduction

Alzheimer’s disease (AD) is the most common form of dementia. It mostly affects people aged 65 and older, progresses slowly and leads to death in an average of nine years after diagnosis. Two hallmarks of Alzheimer’s disease are amyloid plaques and neurofibrillary tangles. Amyloid plaques are extracellular deposits of amyloid-beta peptides (Aβ) derived from the amyloid precursor protein (APP), whereas neurofibrillary tangles (NFTs) are intracellular aggregates of hyperphosphorylated tau protein (hTau). Studies have shown that amyloid deposition leads to tangle formation, neuroinflammation, synaptic dysfunction and neuronal loss^1^. Now we know that Alzheimer’s disease begins decades before the onset of symptoms. However, we need to learn more about the changes that lead to the symptoms and how we can prevent, stop or slow the disease^2^.

Currently, biological data are generated at a higher pace than they are interpreted. Therefore, the challenge is to extract new knowledge from existing data. A meta-analysis combines multiple studies to increase sample size over individual studies. A few studies have conducted a meta-analysis of gene expression data in Alzheimer’s disease. In 2015, Li *et al*. identified 3124 dysregulated genes in the frontal cortex of AD brains from six studies, revealing upregulation of TL4-mediated NF-κB signalling and downregulation of mitochondrial function^3^. Later, Puthiyedth *et al*. found 479 dysregulated genes and two upregulated miR precursors in the entorhinal cortex, hippocampus, middle temporal gyrus, posterior cingulate cortex, and superior frontal gyrus^4^. Recently, Moradifard *et al*. detected 1404 dysregulated genes and 179 dysregulated miRs in eight brain regions from seven studies. They predicted that downregulated miR-30a targets two upregulated genes and six downregulated genes in synaptic plasticity^5^. A large meta-analysis by Patel *et al*. identified 323, 435, 1023 and 828 dysregulated genes in AD temporal lobe, frontal lobe, parietal lobe and cerebellum, respectively, with three genes downregulated and four genes upregulated in all regions^6^. Finally, an integration of genomics and genetics data by Bihlmeyer *et al*. most significantly enriched calcium signalling pathway^7^.

**Figure 1:**
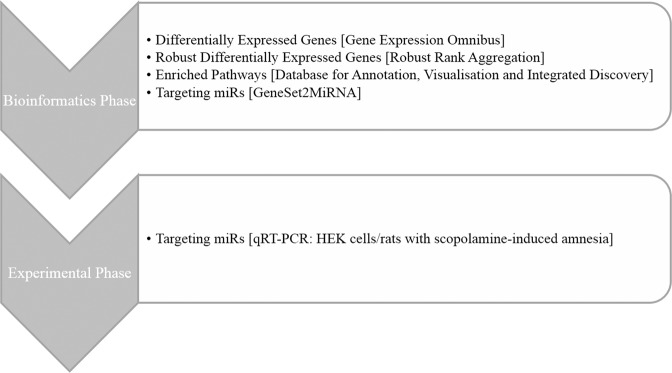
Study flowchart.

We took the initiative to understand the biological meaning behind differentially expressed genes in the hippocampus of brains with Alzheimer’s disease. Our research comprised both bioinformatics and experimental phases (Fig. 1). In the bioinformatics phase, we collected lists of differentially expressed genes (DEGs) to find 1) robust DEGs between studies, 2) pathways enriched by DEGs, 3) miRs targeting DEGs, and 4) regional/cell specificity of DEGs. In the experimental phase, we validated a miR to regulate most of its predicted target genes in HEK cells. We also confirmed the dysregulation of the miR in the hippocampus of brains of rats with scopolamine-induced amnesia as a model of Alzheimer’s disease. This study shows how the exploration of existing data could lead to novel findings.

## Results

### Differentially expressed genes

**Figure 2:**
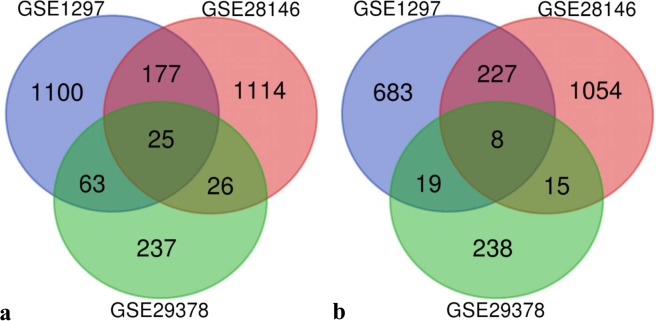
Overlap of **a)** upregulated genes and **b)** downregulated genes between studies. Developed by Van de Peer Lab. But Van de Peer Lab should be a hyperlink to: http://bioinformatics.psb.ugent.be/webtools/Venn/.

We searched GEO to find DEGs in the hippocampus of brains with AD. We found three studies on the CA1 of the hippocampus (Table 1)^8–10^. Please note GSE1297 and GSE28146 used the same subjects with two differences: First, GSE1297 and GSE28146 examined freshly frozen (FF) tissue and formalin-fixed paraffin-embedded (FFPE) block, respectively. Second, GSE1297 and GSE28146 analysed hand-dissected mixed tissue and laser-dissected grey matter, respectively. Both GSE1297 and GSE28146 had three groups of control, incipient AD and overall AD, while GSE29378 comprised two groups of control and AD. Therefore, we only looked at overall AD group. In general, AD brains had an average Braak stage of V-VI. Blalock *et al*. and Miller *et al*. found DEGs in AD by Pearson correlation test and Student’s *t*-test, respectively. We used a Venn diagram to see whether gene lists had an overlap (Fig. 2). We found 25 upregulated genes and eight downregulated genes common between all three studies that we discuss further (Supplementary Table S1). We noticed that the overlap was more than expected by chance (Supplementary Table S2). In total, 2742 upregulated genes and 2244 downregulated genes were unique.

**Table 1:** Studies on the CA1 of the hippocampus of brains with Alzheimer’s disease.

GEO Accession	Contributors	Samples	Platforms	DEGs
GSE1297	Blalock *et al*., 2004^8^	9 controls, 22 AD	Affymetrix HG-U133A	1365 upregulated, 937 downregulated
GSE28146	Blalock *et al*., 2011^9^	8 controls, 22 AD	Affymetrix HG-U133 v2	1342 upregulated, 1304 downregulated
GSE29378	Miller *et al*., 2013^10^	16 controls, 17 AD	Illumina HumanHT-12 v3 Expression BeadChips	351 upregulated, 280 downregulated

### Robust differentially expressed genes

We used RRA to find robust DEGs between studies. We found that 51 upregulated genes and 44 downregulated genes were robust (Supplementary Table S3, Table 2). Please note a robust item may not be common between all lists. In this case, five out of 51 robust upregulated genes, *AQP1*, *DTNA*, *GFAP*, *SERPINA3* and *SPARC*, and two out of 44 robust downregulated genes, *RIMS2* and *SLC6A1*, were common between all three studies (Fig. 2). Two robust dysregulated genes, *FBXO32* and *ANAPC13*, were dysregulated not only in the HIP but also the EC, MTG, PC, and SFG in AD. 40 out of 51 robust upregulated genes were glia-specific, while 34 out of 44 robust downregulated genes were neuron-specific (Supplementary Table S4). Notably, the top four robust upregulated genes were astrocytic, while the top five robust downregulated genes were neuronal. We asked whether the pattern of upregulation of astrocytic genes and downregulation of neuronal genes is the effect of astrocytic activation and neuronal loss seen in Alzheimer’s disease. Therefore, we checked *GFAP* as an astrocytic marker and *RBFOX3* as a neuronal marker. We found upregulation of *GFAP* in all three studies and downregulation of *RBFOX3* in none. Thus, we suggest that, unlike the upregulation of astrocytic genes, the downregulation of neuronal genes is the effect of the disease.

**Table 2:** Top five robust differentially expressed genes.

Gene Symbol	Full Name	Adj. *P*-value	Log2 FC
**Robust Upregulated**			
*SPARC*	secreted protein acidic and cysteine rich	8.0 E-5	5.9 E-1
*BOC*	BOC cell adhesion associated, oncogene regulated	1.9 E-3	5.5 E-1
*S100A6*	S100 calcium binding protein A6	2.9 E-3	6.3 E-1
*SMAD9*	SMAD family member 9	4.6 E-3	7.2 E-1
*CYLC1*	cylicin 1	5.9 E-3	7.0 E-1
**Robust Downregulated**			
*WFDC1*	WAP four-disulfide core domain 1	1.2 E-3	−5.2 E-1
*THYN1*	thymocyte nuclear protein 1	4.0 E-3	−1.6 E-1
*KALRN*	kalirin RhoGEF kinase	4.0 E-3	−4.6 E-1
*TNNI3K*	TNNI3 interacting kinase	4.0 E-3	−3.0 E-1
*RIMS2*	regulating synaptic membrane exocytosis 2	4.6 E-3	−3.9 E-1

### Pathways enriched by differentially expressed genes

We used DAVID to find pathways enriched by robust DEGs. Upregulated genes enriched endocytosis, while downregulated genes enriched Parkinson’s disease, oxidative phosphorylation, glycolysis, and Huntington’s disease. However, these pathways did not pass multiple test correction. Also, we used GS2M to find miRs targeting robust DEGs. We predicted miR-459 and miR-591 for upregulated genes, plus miR-459 and miR-218 for downregulated genes. Similarly, these results were not statistically significant. Therefore, we speculated that the number of robust DEGs was not sufficient for statistical analysis.

**Figure 3:**
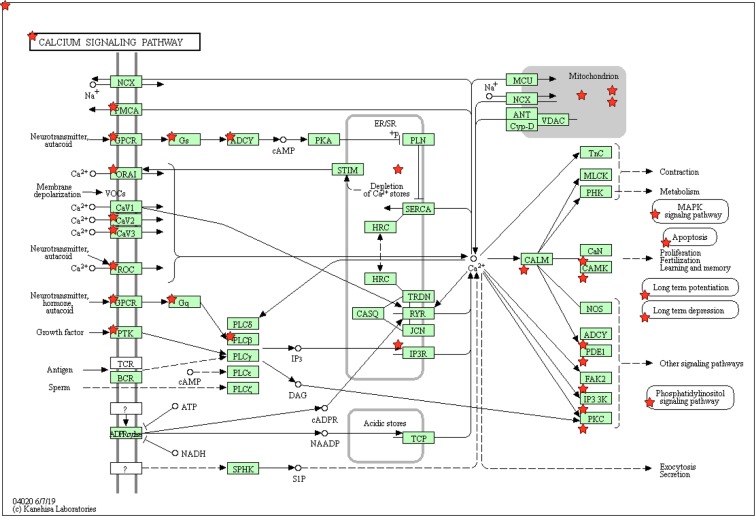
Calcium signalling pathway enriched by 48 downregulated genes encoding 23 proteins marked by stars. Reproduced with copyright permission from KEGG^54,55^.

Then, we used DAVID to find pathways enriched by all DEGs. Upregulated and downregulated genes enriched 11 and 13 pathways, respectively. Since two studies were on the same subjects, we used LOOCV to omit the bias towards these two studies: We excluded three studies one by one to assess whether the pathways were still statistically significant. To our surprise, all 11 pathways enriched by upregulated genes and 12 out of 13 pathways enriched by downregulated genes lost statistical significance. Only calcium signalling pathway, including 48 downregulated genes, was consistent between all three studies (Supplementary Table S5). Figure 3 shows 23 proteins encoded by 48 downregulated genes in the calcium signalling pathway (Supplementary Table S6). Please note we analysed GSE84422^11^ as a newer and larger dataset in which genes negatively correlated with five cognitive or neuropathological traits, Braak, CDR, CERAD, NPrSum and NTrSum, significantly enriched calcium signalling pathway. We also analysed GSE67333^12^ as an RNA sequencing dataset in which downregulated genes significantly enriched calcium signalling pathway. These genes include *ADRA1D*, *ADRB3* and *TRHR* coding for GPCR, plus *P2RX2* coding for ROC.

### miRs targeting differentially expressed genes

We used GS2M to find miRs targeting 48 downregulated genes in the calcium signalling pathway. We predicted seven miRs for some genes (Table 3). We focused on miR-129 because it had the highest number of predicted target genes. We showed the bindings sites of miR-129 on its predicted target genes in Supplementary Table S7^13–15^.

**Table 3:** miRs predicted to target downregulated genes in the calcium signalling pathway.

miR	Adj. *P*-value	Target Genes
hsa-miR-330-3p	0.01	*ATP2B1, ATP2B2, CHP, GRM5, PLCB1, PRKCB*
hsa-miR-129-5p	0.01	*ADCY2, ATP2B1, ATP2B3, CALM1, CAMK2D, CAMK4, PDGFRA, PPP3CA, PRKCB*
hsa-miR-614	0.01	*ADCY1, CALM1, CAMK2A, GRM5, PLCB1*
hsa-miR-181b	0.01	*ADCY1, ATP2A2, ATP2B1, ATP2B2, CALM1, GRM5, PDGFRA*
hsa-miR-30c	0.01	*ADRB1, ATP2A2, ATP2B1, ATP2B2, CAMK2D, GRM5, PPID, PPP3CA*
hsa-miR-181d	0.02	*ADRB1, ATP2A2, ATP2B1, ATP2B2, CAMK2D, GRM5, PPID, PPP3CA*
hsa-miR-30a	0.03	*ATP2A2, ATP2B1, ATP2B2, CAMK2D, GRM5, PPID, PPP3CA*

**Figure 4:**
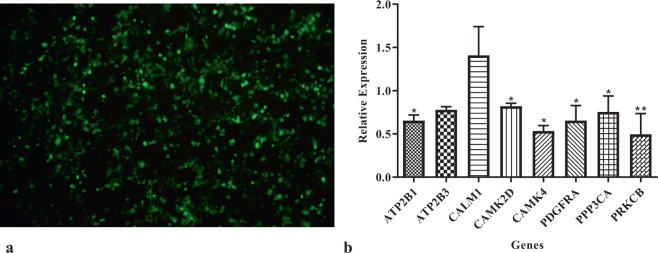
(**a**) Transfection of miR-129 into HEK cells. (**b)** Significant downregulation of six out of nine predicted target genes 48 h after transfectin of miR-129. *ADCY2* showed no expression in HEK cells. *P-values < 0.05, **P-values < 0.01.

First, we assessed whether miR-129 regulates its predicted target genes. When we overexpressed miR-129 in HEK cells, six out of nine predicted target genes were significantly downregulated (Fig. 4).

**Figure 5:**
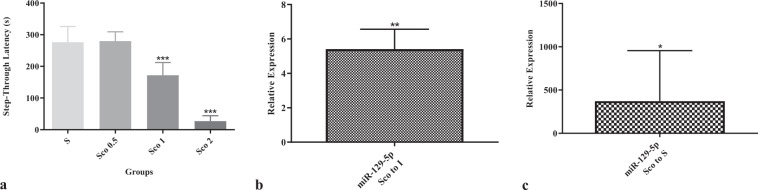
(**a**) Significant decrease in step-through latency as a measure of memory retrieval after administration of 0.5, 1 and 2 mg/kg scopolamine to rats. (**b,c)** Significant upregulation of miR-129 in the hippocampus of brains of rats treated with 2 mg/kg scopolamine compared to both intact and saline controls. I: intact, S: saline, Sco: scopolamine, *P-values < 0.05, **P-values < 0.01, ***P-values < 0.001.

Second, we asked whether miR-129 is upregulated in Alzheimer’s disease, given that its target genes are downregulated. We used rats with scopolamine-induced amnesia as a model of Alzheimer’s disease. We found that miR-129 is significantly upregulated in the hippocampus of brains of rats with scopolamine-induced amnesia (Fig. 5).

## Discussion

We analysed about 2700 upregulated genes and 2200 downregulated genes in the CA1 of the hippocampus of brains with Alzheimer’s disease. We found some dysregulated genes we discuss further. *AQP1*, *DTNA*, *GFAP*, and *SERPINA3* were robust upregulated genes common between all three studies (Fig. 2). *AQP1* encodes an integral membrane protein that functions as a water channel protein. Studies suggest that AQP1-expressing astrocytes may play a role in the formation of amyloid plaques in Alzheimer’s disease^16^. *DTNA* codes for a member of the dystrophin family with biased expression in the brain^17^. *GFAP*, glial fibrillary acidic protein, encodes a major intermediate filament protein of astrocytes. It is elevated in the cerebrospinal fluid of AD brains^18^. SERPINA3 is a plasma protease inhibitor and a member of the serine protease inhibitor class. Studies show that SERPINA3 promotes amyloid deposition^19^, tangle formation and neuronal death^20^ in Alzheimer’s disease. It is elevated in the cerebrospinal fluid^21^ and serum^20^ of AD patients.

*FBXO32* was a robust upregulated gene not only in AD hippocampus but also the entorhinal cortex, middle temporal gyrus, posterior cingulate cortex, and superior frontal gyrus. *FBXO32* codes for a member of the F-box protein family that function in phosphorylation-dependent ubiquitination. Recent studies show that FBXO32 activates NF-κB signalling pathway by ΙκΒα degradation during inflammation^22^. We suppose that it may explain the upregulation of FBXO32 in several brain regions in Alzheimer’s disease.

*KALRN* was a top robust downregulated gene. Kalirin is a Rho guanine exchange factor that promotes remodelling of the actin filaments, leading to dendritic spine formation^23^. It is downregulated in the hippocampus of AD brains^24^.

*RIMS2 and SLC6A1* were robust downregulated genes common between all three studies (Fig. 2). RIMS2 is a presynaptic RAB3 interacting molecule involved in calcium-induced neurotransmitter release^25^. GAT1, encoded by *SLC6A1*, is the major GABA transporter in the brain. It transfers GABA from the synaptic cleft to the presynaptic terminal. GAT1 knockout mice show impairment in hippocampal-dependent learning and memory^26^.

We validated miR-129 to regulate four genes that play a role in synaptic plasticity, namely *CAMK2D*, *CAMK4*, *PPP3CA*, and *PRKCB* coding for CaMKII, CaMKIV, CaN, and PKC, respectively (Fig. 4). Two forms of synaptic plasticity are long-term potentiation (LTP) and long-term depression (LTD). CaMKII induces LTP, while CaN triggers LTD. In LTP, CaMKII and PKC phosphorylate existing AMPARs to enhance their activity^27,28^ and modulate the insertion of additional AMPARs into the postsynaptic membrane^29,30^. Also, CaMKIV activates CREB^31^, which induces the transcription of genes associated with synaptic plasticity. In LTD, PKC phosphorylates AMPARs leading to their endocytosis^32^. Studies confirm that CaMKII^33^, CaMKIV^34^, and PKC^35^ decrease in the hippocampus of AD brains, while CaN increases^36^. It is consistent with the fact that the balance between LTP and LTD is favoured to LTD in Alzheimer’s disease. Several studies show that overexpression of CaMKII^37^, CaMKIV^38^, and PKC^39^, as well as inhibition of CaN^40^, make improvements in animal models of Alzheimer’s disease.

We also confirmed the upregulation of miR-129 in the hippocampus of brains of rats with scopolamine-induced amnesia as a model of Alzheimer’s disease (Fig. 5). miR-129 has a high expression in the brain^41^ at synapses^42^. It has over six hundred target genes, enriched in the estrogen signalling pathway, axon guidance, thyroid hormone signalling pathway, and neurotrophic signalling pathway^43^. It targets *MAPK1* coding for ERK1/2^44^. It represses a potassium channel in an mTOR-dependent manner, leading to dendritic excitability^45^. We also found the mTOR signalling pathway enriched by upregulated genes: *CAB39L*, *IGF1*, *MAPK1*, *RHEB*, *RICTOR*, *RPS6KA1*, *STK11*, *ULK2*, *VEGFB*, and *VEGFC*. Relevantly, a recent meta-analysis predicted miR-30a to target *CAMK2B*, *CAMK4*, *MAPK1*, *PPP3CB*, *PPP3R1*, and *RSP6KA2* in LTP in Alzheimer’s disease^5^. Therefore, we believe miR-129 and its target genes play a role in synaptic plasticity in Alzheimer’s disease.

It should be noted that scopolamine is a muscarinic receptor blocker inducing many of cellular and molecular changes in Alzheimer’s disease including cholinergic dysfunction, Aβ and tau pathology, oxidative stress, mitochondrial dysfunction, neuroinflammation and apoptosis^46^. We assessed memory retrieval by a step-through passive avoidance task in which the animal avoids an aversive stimulus after learning the association between a dark chamber and a mild electrical foot shock^47^. This model was specifically useful for us as we were investigating the brain region, pathways and genes underlying learning and memory.

In conclusion, we reached from thousands of dysregulated genes to tens of robust dysregulated genes, tens of dysregulated genes enriched in a pathway, and a handful of dysregulated genes targeted by a miR. We noticed that downregulated genes highlight synaptic function. We suggest that future research should investigate the possible role of miR-129 in synaptic plasticity and Alzheimer’s disease. Next step would be to confirm the interaction of miR-129 and its target genes by dual-luciferase reporter assay. It is also needed to confirm the upregulation of miR-129 in the hippocampus of humans with Alzheimer’s disease. Further work could look at the inhibition of miR-129 in models of Alzheimer’s disease. We recommend following our framework to gain insight into potential biomarkers and drug targets for diagnosis and treatment of diseases.

## Methods

### Bioinformatics Phase

#### DEGs

Gene Expression Omnibus (GEO) is a free public database containing MIAME-compliant data^48,49^. We searched GEO DataSets with two keywords of Alzheimer and hippocampi/hippocampus, plus three filters of *Homo sapiens*, Series, and Expression profiling by array. We found three studies on the CA1 of the hippocampus. We obtained lists of DEGs from supplementary materials of papers^8–10^. We considered DEGs with a *p*-value < 0.05 as statistically significant. FDR was 0.19, 0.16 and N/A in three studies, respectively^8–10^.

#### Robust DEGs

Unlike Rank Aggregation (RA) method that finds the closest list to the input lists, Robust Rank Aggregation (RRA) method provides a relevant list of even irrelevant and incomplete input lists^50^. We combined three lists of gene symbols into a tab-delimited text file. We wrote a code to read and aggregate three lists of arbitrary lengths in a tab-delimited text file (Supplementary Table S8)^51^. We considered robust DEGs with a Bonferroni-corrected *p*-value < 0.05 as statistically significant.

#### Enriched Pathways

The Database for Annotation, Visualisation, and Integrated Discovery (DAVID) comprises a set of functional annotation tools to understand the biological meaning behind a gene list^52,53^. We submitted and combined three lists of probe IDs in DAVID 6.7. Only 0.6% of probe IDs were not mapped to DAVID IDs. We selected KEGG-pathway from functional annotation tools^54,55^. We considered pathways with a Benjamini-corrected *p*-value < 0.05 as statistically significant. We used leave-one-out cross-validation (LOOCV) to reduce the number of pathways: We excluded each list, combined two others and evaluated the statistical significance of the pathways.

#### Targeting miRs

GeneSet2miRNA (GS2M) finds miRs targeting a gene list^56^ using the Predicted Targets component of miRecords^57^, which integrates 11 prediction programs^58^. We submitted a list of gene symbols updated by HUGO Gene Nomenclature Committee (HGNC)^59^ to GS2M on BioProfiling. All gene symbols were recognised. We considered miRs with a Mont Carlo-corrected *p*-value < 0.05 as statistically significant.

#### Regional/Cell Specificity

We obtained a list of 26 DEGs common between the entorhinal cortex (EC), hippocampus (HIP), middle temporal gyrus (MTG), posterior cingulate cortex (PC), and superior frontal gyrus (SFG) in AD from GSE5281^60^ for the analysis of regional specificity.

We used a list of 8166, 2995, 1231 and 1926 genes assigned to neuron, astrocyte, microglia, and oligodendrocyte, respectively, from GSE29378^10^ for the analysis of cell specificity.

## Experimental Phase

### qRT-PCR

We had three biological replicates for cells and animals, plus two technical replicates in qPCR. We used *SNORD47* and *ACTB* as internal controls for miR and mRNA expression analyses, respectively (Supplementary Table S9). We extracted total RNA using the Hybrid-R kit (GeneAll, Korea). We performed qPCR on the StepOnePlus system (Applied Biosystems, USA). We used the Relative Expression Software Tool (REST)^61^ for expression analysis.

### Animals

The subjects were male Wistar rats weighing 220-250 g. They were housed in groups of four per cage under a standard condition of 12 h light/dark cycle at 22 ± 2 °C. Food and water were available ad libitum. We performed all experiments between 9:00 and 15:00. We made all efforts to minimise the number of animals used and their suffering. We carried out all experiments in accordance with institutional guidelines for animal care and use. The Research and Ethics Committee of the College of Science, University of Tehran approved all experiments.

### Passive Avoidance Learning

We used the step-through passive avoidance apparatus (BorjSanat, Iran) to evaluate memory retrieval in animals. The apparatus was a 20×20×30 cm Plexiglas box divided into two equal chambers, one white and the other dark, connected via a guillotine door. The floor of the dark chamber was stainless steel grids connected to a stimulator to produce an electrical shock (50 Hz, 3 s, 1 mA). On the training day, we allowed animals to habituate in the experimental room. After 1 h, we gently placed each animal in the white chamber. After 5 s, we opened the guillotine door and allowed the animal to enter the dark chamber. Once the animal crossed with all four paws to the dark chamber, we closed the door and took the animal into its home cage. After 30 min, we repeated the procedure, but this time, as soon as the animal crossed to the dark chamber, we closed the door and delivered a foot shock to the animal via the floor of the dark chamber. Two minutes later, we retested the animal and measured the latency to enter the dark chamber. We recorded the successful acquisition of the passive avoidance response after 120 s. Immediately after training, all animals received an intraperitoneal injection of 0.5, 1 and 2 mg/kg scopolamine or saline. 24 h after training, we performed the retrieval test with cut off time up to 300 s (Fig. 5). Then immediately, we sacrificed animals and extracted their hippocampal formation.

## Supplementary information

Supplementary Information.

## Data Availability

All supporting data are available as supplementary information. The authors declare no restrictions on data availability.
