# Supplementary material for: A meta-analysis of gene expression data highlights synaptic dysfunction in the hippocampus of brains with Alzheimer’s disease
Source: Sci Rep. 2020 May 20;10:8384. doi: 10.1038/s41598-020-64452-z (PMC7239885; doi:10.1038/s41598-020-64452-z)
Supplement: Supplementary file 1 — Supplementary Information. [file 41598_2020_64452_MOESM1_ESM.docx]

**A meta-analysis of gene expression data highlights synaptic dysfunction in the hippocampus of brains with Alzheimer’s disease**

1. Saeedeh Hosseinian

Department of Biotechnology, College of Science, University of Tehran, Tehran, Iran

saeedeh.hosseinian@gmail.com

2. Ehsan Arefian*

Department of Microbiology, School of Biology, College of Science, University of Tehran, Tehran, Iran

Pediatric Cell Therapy Research Center, Tehran University of Medical Sciences, Tehran, Iran

arefian@ut.ac.ir

3. Hassan Rakhsh-Khorshid

Department of Biochemistry, Faculty of Biological Sciences, Tarbiat Modares University, Tehran, Iran

h.rakhshkhorshid@modares.ac.ir

4. Mehdi Eivani

Neuroscience Lab, Department of Animal Biology, School of Biology, College of Science, University of Tehran, Tehran, Iran

mehdi.eivani@gmail.com

5. Ameneh Rezayof

Neuroscience Lab, Department of Animal Biology, School of Biology, College of Science, University of Tehran, Tehran, Iran

rezayof@khayam.ut.ac.ir

6. Hamid Pezeshk

School of Mathematics, Statistics and Computer Science, College of Science, University of Tehran, Tehran, Iran

School of Biological Sciences, Institute for Research in Fundamental Sciences (IPM), Tehran, Iran

pezeshk@ut.ac.ir

7. Sayed-Amir Marashi

Department of Biotechnology, College of Science, University of Tehran, Tehran, Iran

marashi@ut.ac.ir

**Supplementary Table S1.** Differentially expressed genes common between studies.

| **Upregulated Genes** | | | **Downregulated Genes** |
| --- | --- | --- | --- |
| *AQP1*  *BCL6*  *C1R*  *CD44*  *DTNA*  *EGFR*  *EMP1*  *ERBB2IP*  *GADD45B* | *GFAP*  *ID4*  *MCL1*  *MYBPC1*  *NAV2*  *NFE2L1*  *PPFIA1*  *PRCP*  *PRKX* | *SERPINA3*  *SLC14A1*  *SOD2*  *SPARC*  *TGFBR3*  *UNG*  *ZFP36L2* | *B3GALT2*  *CAMKK2*  *KCNQ2*  *MEG3*  *PCP4*  *PLCB1*  *RIMS2*  *SLC6A1* |

**Supplementary Table S2.** Statistical significance of the overlap of differentially expressed genes between studies.

| **Lists: Number of Unique Genes** | **Pairs: Number of Common Genes** | [**Statistical Significance**](http://nemates.org/MA/progs/overlap_stats.html) |
| --- | --- | --- |
| **Upregulated Genes** | | |
| GSE1297: 1365 | GSE1297 and GSE28146: 177 | RF 1.7, *p*-value < 5.1E-13 |
| GSE28146: 1342 | GSE28146 and GSE29378: 26 | RF 1, *p*-value < 0.49 |
| GSE29378: 351 | GSE1297 and GSE29378: 63 | RF 2.3, *p*-value < 2.5E-10 |
| **Downregulated Genes** | | |
| GSE1297: 937 | GSE1297 and GSE28146: 227 | RF 3.3, *p*-value < 3.7E-62 |
| GSE28146: 1304 | GSE28146 and GSE29378: 15 | RF 0.7, *p*-value < 0.111 |
| GSE29378: 280 | GSE1297 and GSE29378: 19 | RF 1.3, *p*-value < 0.166 |

**Supplementary Table S3.** Robust differentially expressed genes.

| **Robust Upregulated Genes** | | | **Robust Downregulated Genes** | | |
| --- | --- | --- | --- | --- | --- |
| **Gene Symbol** | **Adj. *P*-value** | **Log2 FC** | **Gene Symbol** | **Adj. *P*-value** | **Log2 FC** |
| *SPARC* | 8.0 E-5 | 5.9 E-1 | *WFDC1* | 1.2 E-3 | -5.2 E-1 |
| *BOC* | 1.9 E-3 | 5.5 E-1 | *THYN1* | 4.0 E-3 | -1.6 E-1 |
| *S100A6* | 2.9 E-3 | 6.3 E-1 | *KALRN* | 4.0 E-3 | -4.6 E-1 |
| *SMAD9* | 4.6 E-3 | 7.2 E-1 | *TNNI3K* | 4.0 E-3 | -3.0 E-1 |
| *CYLC1* | 5.9 E-3 | 7.0 E-1 | *RIMS2* | 4.6 E-3 | -3.9 E-1 |
| *PTPN13* | 5.9 E-3 | 4.3 E-1 | *NGFRAP1* | 8.0 E-3 | -1.0 E-1 |
| *GEM* | 5.9 E-3 | 2.7 E-1 | *ARHGAP20* | 8.0 E-3 | -5.6 E-1 |
| *GFAP* | 7.0 E-3 | 4.7 E-1 | *DEPDC5* | 8.0 E-3 | -2.7 E-1 |
| *HBEGF* | 8.9 E-3 | 3.2 E-1 | *SLC6A1* | 8.1 E-3 | -3.8 E-1 |
| *MALAT1* | 8.9 E-3 | 2.4 E-1 | *NDUFA2* | 8.8 E-3 | -1.6 E-1 |
| *PFKFB3* | 8.9 E-3 | 3.9 E-1 | *TM2D3* | 1.1 E-2 | -4.3 E-1 |
| *DDR1* | 1.1 E-2 | 4.4 E-1 | *TPX2* | 1.2 E-2 | -6.8 E-1 |
| *ACAP1* | 1.1 E-2 | 3.3 E-1 | *SEPT5* | 1.2 E-2 | -5.9 E-1 |
| *AHNAK* | 1.4 E-2 | 3.9 E-1 | *MRPL15* | 1.2 E-2 | -5.0 E-1 |
| *FOXO1* | 1.4 E-2 | 5.5 E-1 | *TUBG2* | 1.6 E-2 | -2.4 E-1 |
| *SNORA68* | 1.4 E-2 | 6.7 E-1 | *NDUFA4* | 1.6 E-2 | -2.5 E-1 |
| *TEX2* | 1.4 E-2 | 3.2 E-1 | *ZNF362* | 1.6 E-2 | -2.7 E-1 |
| *HIST1H1C* | 1.7 E-2 | 6.0 E-1 | *ENO2* | 1.8 E-2 | -6.6 E-1 |
| *TMC7* | 1.7 E-2 | 1.5 | *AKAP11* | 2.0 E-2 | -4.1 E-1 |
| *SERPINA3* | 1.7 E-2 | 8.5 E-1 | *THY1* | 2.0 E-2 | -2.0 E-1 |
| *IL10RA* | 2.0 E-2 | 3.9 E-1 | *ZNF134* | 2.0 E-2 | -2.7 E-1 |
| *WWOX* | 2.0 E-2 | 4.4 E-1 | *CSPG5* | 2.0 E-2 | -3.1 E-1 |
| *KPNA4* | 2.0 E-2 | 3.1 E-1 | *FRG1* | 2.0 E-2 | -2.7 E-1 |
| *ELF1* | 2.3 E-2 | 4.0 E-1 | *PDHA1* | 2.1 E-2 | -3.0 E-1 |
| *AFF4* | 2.3 E-2 | 1.7 E-1 | *AP2B1* | 2.4 E-2 | -3.5 E-1 |
| *CABC1* | 2.3 E-2 | 2.6 E-1 | *UBE2M* | 2.4 E-2 | -1.8 E-1 |
| *DTNA* | 2.4 E-2 | 6.1 E-1 | *VPS13B* | 2.4 E-2 | -1.8 E-1 |
| *HERC5* | 2.6 E-2 | 5.7 E-1 | *ANAPC13* | 2.8 E-2 | -1.5 E-1 |
| *DOK1* | 2.6 E-2 | 3.6 E-1 | *RAP1GAP* | 2.8 E-2 | -6.8 E-1 |
| *MAB21L2* | 2.9 E-2 | 5.6 E-1 | *GNG3* | 3.0 E-2 | -4.0 E-1 |
| *MDM2* | 2.9 E-2 | 4.0 E-1 | *GRPEL1* | 3.1 E-2 | -4.3 E-1 |
| *TPST1* | 2.9 E-2 | 6.6 E-1 | *ATP5J2* | 3.1 E-2 | -3.2 E-1 |
| *RGS1* | 2.9 E-2 | 1.4 | *MAPRE3* | 3.5 E-2 | -2.5 E-1 |
| *FBXO32* | 3.1 E-2 | 6.1 E-1 | *ATP6AP2* | 3.5 E-2 | -4.9 E-1 |
| *MYO1F* | 3.2 E-2 | 7.5 E-1 | *FAM5C* | 3.5 E-2 | -2.3 E-1 |
| *SH3TC1* | 3.2 E-2 | 7.9 E-1 | *NDUFB5* | 3.9 E-2 | -1.3 E-1 |
| *CMTM4* | 3.2 E-2 | 2.2 E-1 | *C1ORF57* | 3.9 E-2 | -2.5 E-1 |
| *ERBB2IP* | 3.5 E-2 | 5.0 E-1 | *ADAMTS8* | 3.9 E-2 | -4.2 E-1 |
| *PAX6* | 3.5 E-2 | 4.6 E-1 | *PDCD5* | 4.3 E-2 | -8.7 E-1 |
| *ADCK2* | 3.5 E-2 | 1.1 | *CXCL14* | 4.3 E-2 | -6.7 E-1 |
| *RAMP1* | 3.5 E-2 | 2.3 E-1 | *ERLEC1* | 4.3 E-2 | -4.6 E-1 |
| *ERF* | 3.8 E-2 | 4.3 E-1 | *PJA1* | 4.7 E-2 | -2.0 E-1 |
| *HSPB2* | 3.8 E-2 | 4.2 E-1 | *HK1* | 4.7 E-2 | -2.9 E-1 |
| *KDM5B* | 4.1 E-2 | 6.4 E-1 | *TMEM145* | 4.7 E-2 | -5.9 E-1 |
| *AQP1* | 4.4 E-2 | 4.3 E-1 |  |  |  |
| *SLC19A1* | 4.4 E-2 | 7.5 E-1 |  |  |  |
| *SAMD4B* | 4.4 E-2 | 3.0 E-1 |  |  |  |
| *CXCR4* | 4.4 E-2 | 8.5 E-1 |  |  |  |
| *SPAG1* | 4.7 E-2 | 3.4 E-1 |  |  |  |
| *ANKRD26* | 4.7 E-2 | 5.9 E-1 |  |  |  |
| *C9ORF89* | 4.7 E-2 | 2.7 E-1 |  |  |  |

**Supplementary Table S4.** Cell specificity of robust differentially expressed genes.

| **Glial Robust Upregulated Genes** | | | | **Neuronal Robust Downregulated Genes** | | |
| --- | --- | --- | --- | --- | --- | --- |
| **Astrocytic** | | **Microglial** | **Oligodendrocytic** |  |  |  |
| *AHNAK*  *APQ1*  *BOC*  *C9ORF89*  *CMTM4*  *CXCR4*  *DDR1*  *DTNA*  *ELF1*  *ERBB2IP*  *FBXO32*  *FOXO1*  *GEM*  *GFAP* | *HIST1H1C*  *HSPB2*  *IL10RA*  *PAX6*  *PFKFB3*  *RAMP1*  *S100A6*  *SERPINA3*  *SH3TC1*  *SMAD9*  *SPAG1*  *SPARC*  *TEX2*  *TPST1* | *DDR1*  *ELF1*  *HSPB2*  *MYO1F*  *PAX6*  *RGS1*  *SH3TC1*  *SPAG1* | *HERC5*  *TMC7*  *HBEGF*  *FBXO32* | *AKAP11*  *ANAPC13*  *AP2B1*  *ARHGAP20*  *ATP5J2*  *ATP6AP2*  *C1ORF57*  *CSPG5*  *ENO2*  *FAM5C*  *FRG1*  *GNG3*  *GRPEL1*  *HK1* | *KALRN*  *MAPRE3*  *MRPL15*  *NDUFA4*  *NGFRAP1*  *PDCD5*  *PDHA1*  *PJA1*  *RAP1GAP*  *THY1*  *THYN1*  *TM2D3*  *TMEM145*  *TNNI3K* | *TUBG2*  *UBE2M*  *VPS13B*  *WFDC1*  *ZNF134*  *ZNF362* |

**Supplementary Table S5.** Leave-one-out cross-validation for pathways enriched by differentially expressed genes.

| **Term** | **Adj. *P*-value** | **GSE1297 out**  **Adj. *P*-value** | **GSE28146 out**  **Adj. *P*-value** | **GSE29378 out**  **Adj. *P*-value** |
| --- | --- | --- | --- | --- |
| **Upregulated Genes** | | | | |
| Pathways in cancer | 1.6 E-5 | 4.3 E-1 | 1.3 E-5 | 8.2 E-5 |
| Focal adhesion | 1.6 E-3 | 7.5 E-1 | 2.4 E-2 | 7.5 E-4 |
| Adherens junction | 7.5 E-3 | - | 3.7 E-3 | 5.9 E-3 |
| Colorectal cancer | 1.1 E-2 | 5.3 E-1 | 1.7 E-2 | 2.0 E-2 |
| Prostate cancer | 2.4 E-2 | 5.3 E-1 | 1.9 E-2 | 1.7 E-2 |
| ARVC | 2.1 E-2 | 5.8 E-1 | 4.6 E-2 | 3.3 E-2 |
| Dilated cardiomyopathy | 3.0 E-2 | 5.4 E-1 | 1.0 E-1 | 6.8 E-2 |
| MAPK signalling pathway | 3.8 E-2 | 4.5 E-1 | 4.8 E-2 | 7.8 E-2 |
| Notch signalling pathway | 4.5 E-2 | 3.8 E-1 | 8.0 E-2 | 2.6 E-2 |
| Apoptosis | 4.3 E-2 | 5.9 E-1 | 2.0 E-1 | 2.4 E-2 |
| TGF-beta signalling pathway | 4.3 E-2 | 5.0 E-1 | 6.9 E-2 | 2.4 E-2 |
| **Downregulated Genes** | | | | |
| Parkinson’s disease | 1.0 E-13 | 3.2 E-1 | 4.1 E-20 | 1.7 E-21 |
| Oxidative phosphorylation | 5.3 E-13 | 4.5 E-1 | 3.4 E-19 | 1.9 E-21 |
| Alzheimer’s disease | 4.6 E-13 | 1.6 E-1 | 2.3 E-17 | 5.7 E-20 |
| Huntington’s disease | 3.6 E-13 | - | 2.1 E-17 | 6.4 E-20 |
| Long-term potentiation | 9.7 E-4 | 1.5 E-1 | 9.2 E-2 | 7.4 E-2 |
| Cardiac muscle contraction | 3.1 E-3 | - | 1.5 E-3 | 2.3 E-3 |
| Calcium signalling pathway | 3.2 E-3 | 2.8 E-1 | 1.2 E-2 | 6.7 E-2 |
| Glycolysis/Gluconeogenesis | 8.0 E-3 | 2.1 E-1 | 5.8 E-3 | 3.9 E-3 |
| Ala, Asp and Glu metabolism | 1.2 E-2 | - | 2.6 E-2 | 9.1 E-3 |
| Citrate cycle (TCA cycle) | 1.2 E-2 | 3.2 E-1 | 2.3 E-1 | 5.5 E-5 |
| Oocyte meiosis | 1.7 E-2 | 2.3 E-1 | 6.6 E-2 | 3.0 E-2 |
| Axon guidance | 2.4 E-2 | 1.5 E-1 | 2.1 E-1 | 2.9 E-1 |
| Pyruvate metabolism | 3.4 E-2 | 2.4 E-1 | 4.4 E-2 | 1.5 E-2 |

**Supplementary Table S6.** Downregulated genes in the calcium signalling pathway.

| **Protein** | **Gene** | **Protein** | **Gene** | **Protein** | **Gene** |
| --- | --- | --- | --- | --- | --- |
| ADCY | *ADCY1* | CaV2 | *CACNA1A* | IP3 3K | *ITPKA* |
|  | *ADCY2* |  | *CACNA1B* | IP3R | *ITPR1* |
| ANT | *SLC25A5* | CaV3 | *CACNA1I* | ORAI | *TRPC1* |
|  | *SLC25A5P8* | Cyp-D | *PPID* | PDE1 | *PDE1A* |
|  | *SLC25A6* | FAK2 | *PTK2B* |  | *PDEAB* |
| CALM | *CALM1* | GPCR | *ADORA2B* | PKC | *PRKCB* |
|  | *CALM2* |  | *ADRA1B* | PLCB | *PLCB1* |
|  | *CALM3* |  | *ADRB1* | PMCA | *ATP2B1* |
| CAMK | *CAMK2A* |  | *CHRM1* |  | *ATP2B2* |
|  | *CAMK2D* |  | *GRM5* |  | *ATP2B3* |
|  | *CAMK4* |  | *HTR2A* | PTK | *PDGFRA* |
| CaN | *CHP* |  | *HTR2C* | SERCA | *ATP2A2* |
|  | *PPP3CA* | Gq | *GNA14* |  | *GRIN2D* |
|  | *PPP3CB* |  | *GNAQ* | VDAC | *VDAC1* |
|  | *PPP3CC* | Gs | *GNAL* |  | *VDAC1P1* |
|  |  |  | *GNAS* |  | *VDAC3* |

**Supplementary Table S7.** Binding sites of miR-129 on its predicted target genes. Obtained from TargetScan^13^, miRDB^14^ and microRNA.org^15^.

| **Target Gene** | **3’ UTR Length** | **Seed Location** | |
| --- | --- | --- | --- |
| *ADCY2* | 3210 | 2611  3043 | 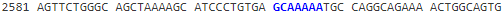  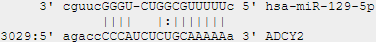 |
| *ATP2B1* | 2952 | 2517 | 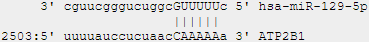 |
| *ATP2B3* | 2631 | 482  2506 | 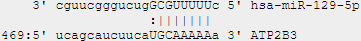  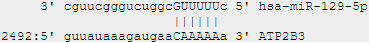 |
| *CALM1* | 3570 | 67 | 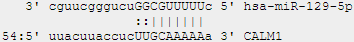 |
| *CAMK2D* | 3524 | 289  1565 | 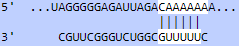  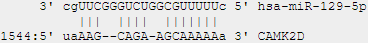 |
| *CAMK4* | 615 | 111  513 | 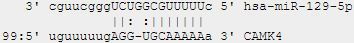  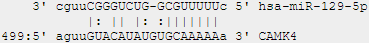 |
| *PDGFRA* | 2973 | 1067 | 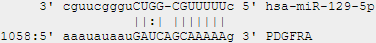 |
| *PPP3CA* | 2435 | 1074  1885 | 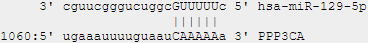  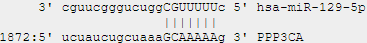 |
| *PRKCB* | 5795 | 822  3175 | 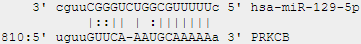  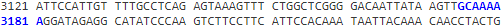 |

**Supplementary Table S8.** The code used for RobustRankAggreg package in R software.

| dat <- read.table(file.choose(), sep = '\t', head = T, na.strings = '')  list1 <- dat[, 1]  list2 <- dat[, 2]  list3 <- dat[, 3]  list1 <- as.character(list1[!is.na(list1)])  list2 <- as.character(list2[!is.na(list2)])  list3 <- as.character(list3[!is.na(list3)])  genelist1 <- list(list1 = list1, list2 = list2, list3 = list3)  ll=c(list1,list2,list3)  N1=length(names(table(ll)))  aggregateRanks(glist = genelist1, N = N1) |
| --- |

**Supplementary Table S9.** Primers used for qRT-PCR.

| **Target** | **5’-RT Primer-3’** | |
| --- | --- | --- |
| *snord47* | GTC GTA TGC AGA GCA GGG TCC GAG GTA TTC GCA CTG CAT ACG ACA ACC TC | |
| *mir-129-5p* | CCA GGT ATG CAG AGC AGG GTC CGA GGT ATC CAT CGC ACG CAT CGC ACT GCA TAC CTG GGC AAG C | |
| **Target** | **5’-Forward Primer-3’** | **5’-Reverse Primer-3’** |
| *snord47* | ATC ACT GTA AAA CCG TTC CA | GAG CAG GGT CCG AGG T |
| *mir-129-5p* | AGC TTT TTG CGG TCT GG |  |
| *ACTB* | CTT CCT TCC TGG GCA TG | GTC TTT GCG GAT GTC CAC |
| *ADCY2* | TTG CGA GTG GGT ATT AAC | CGT GTA TCC GAG GGT C |
| *ATP2B1* | TTG GAG CTG GAG GTG AAG AGG AAG | TCC TGG GCT TTT GCT TTG TTG CG |
| *ATP2B3* | CAC TGG TCA AAG GGA TTA TC | AAG TTG TCA TCG GTC AGG |
| *CALM1* | CGT GCC GTT ACT CGT AG | ATG GTG CGA GCG AAG |
| *CAMK2D* | CCA GGT TCA CGG ACG AGT ATC | CTG ATG ATC CCT AGC AGA AAG C |
